# miR-762 promotes porcine immature Sertoli cell growth via the ring finger protein 4 (*RNF4*) gene

**DOI:** 10.1038/srep32783

**Published:** 2016-09-06

**Authors:** Changping Ma, Huibin Song, Lei Yu, Kaifeng Guan, Pandi Hu, Yang Li, Xuanyan Xia, Jialian Li, Siwen Jiang, Fenge Li

**Affiliations:** 1 Key Laboratory of Pig Genetics and Breeding of Ministry of Agriculture & Key Laboratory of Agricultural Animal Genetics, Breeding and Reproduction of Ministry of Education, Huazhong Agricultural University, Wuhan 430070, PR China; 2 College of Informatics, Huazhong Agricultural University, Wuhan 430070, PR China; 3 The Cooperative Innovation Center for Sustainable Pig Production, Wuhan 430070, PR China

## Abstract

A growing number of reports have revealed that microRNAs (miRNAs) play critical roles in spermatogenesis. Our previous study showed that miR-762 is differentially expressed in immature and mature testes of Large White boars. Our present data shows that miR-762 directly binds the 3′ untranslated region (3′UTR) of ring finger protein 4 (*RNF4*) and down-regulates RNF4 expression. A single nucleotide polymorphism (SNP) in the *RNF4* 3′UTR that is significantly associated with porcine sperm quality traits leads to a change in the miR-762 binding ability. Moreover, miR-762 promotes the proliferation of and inhibits apoptosis in porcine immature Sertoli cells, partly by accelerating DNA damage repair and by reducing androgen receptor (AR) expression. Taken together, these findings suggest that miR-762 may play a role in pig spermatogenesis by regulating immature Sertoli cell growth.

Spermatogenesis is a highly integrated process in which spermatogonia undergo mitotic proliferation once to produce primary spermatocytes, and diploid primary spermatocytes undergo meiosis twice to form haploid spermatids. These haploid spermatids then differentiate into spermatozoa^1^. These events occur in the testicular seminiferous epithelium and require accurate gene expression, which is strictly controlled at the transcriptional and post-transcriptional levels^2^,^3^.

MicroRNAs (miRNAs) are a class of small endogenous noncoding RNAs (approximately 22 nt long). miRNAs bind to the 3′ untranslated region (3′UTR) or coding sequence region (CDS) of their target messenger RNAs (mRNAs), resulting in the degradation (perfect complementarity) or translational inhibition (incomplete match) of the mRNAs^4^,^5^. Accumulating evidence has indicated that miRNAs participate in numerous biological processes, including nervous and cardiovascular system development, cell proliferation and death, and spermatogenesis^6^,^7^,^8^.

We have identified 129 miRNAs that are differentially expressed in immature and mature testes of Large White boars^9^. Several of these miRNAs have already been reported to play essential roles in spermatogenesis. For example, miR-17-92 disruption in the testes of adult mice led to severe testicular atrophy, empty seminiferous tubules, and depressed sperm production, these effects were partly due to the activation of the mTOR signalling pathway^10^. miR-133b is up-regulated in Sertoli cells of Sertoli-cell-only syndrome patients and promotes human Sertoli cell proliferation by targeting the transcription factor GLI family zinc finger 3 (GLI3) and activating Cyclin B1 and Cyclin D1^11^. miR-383 is down-regulated in the testes of infertile men with maturation arrest^12^. miR-383 has been proposed to form a feedback loop with fragile X mental retardation protein (FMRP) to suppress cell proliferation and increase DNA damage, consequently regulating spermatogenesis^12^. The male mice with both miR-449 cluster and miR-34b/c knocked out are sterile and display low sperm counts, low or no motility, and deformed sperm^13^. miR-762 is differentially expressed in immature and mature testes of Large White boars^9^. However, the regulatory mechanism by which miR-762 regulates spermatogenesis is still unknown.

In this study, miR-762 inhibited RNF4 expression. A spontaneous *RNF4* c.* 566 T > C mutation in the *RNF4* 3′UTR was significantly associated with sperm quality traits, possibly due to a change in the binding site number between miR-762 and the *RNF4* 3′UTR. Furthermore, miR-762 promoted immature Sertoli cell proliferation and inhibited apoptosis not via *RNF4* gene directly but possibly by accelerating DNA damage repair and weakening androgen receptor (AR) transcriptional regulatory activity. Our findings suggest that miR-762 may affect spermatogenesis by promoting immature Sertoli cell growth.

## Results

### miR-762 directly targets the *RNF4* 3′UTR

**Figure 1:**
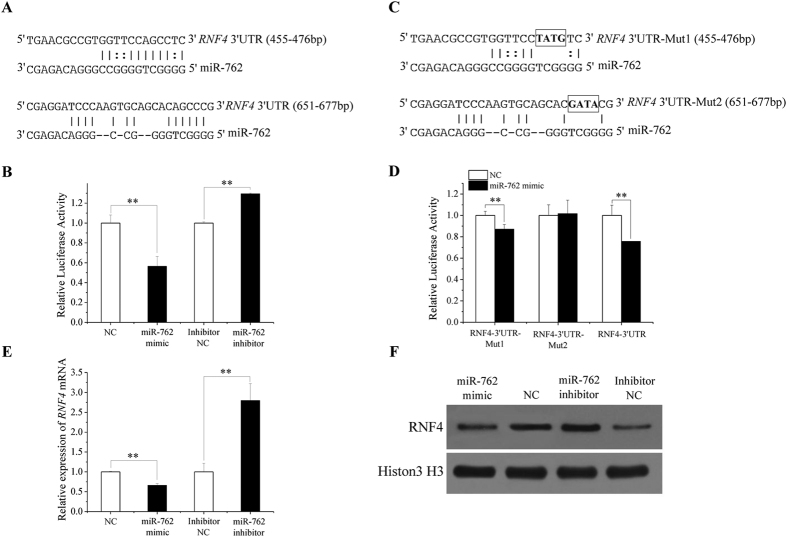
*RNF4* is a target gene of miR-762. (**A**) Two binding sites for miR-762 in the *RNF4* 3′UTR were predicted using RNA22. **(B)** pmirGLO-*RNF4*-3′UTR was co-transfected into ST cells with the miR-762 mimic or NC and the miR-762 inhibitor or inhibitor NC. Whole cellular lysates were obtained 24 h after transfection, and relative luciferase activity was measured. **(C)** pmirGLO-*RNF4*-3′UTR-Mut1 and pmirGLO-*RNF4*-3′UTR-Mut2 were constructed. Black boxes in the seed-matched sites indicated the sequences that were mutated to abolish the interaction between miR-762 and *RNF4* 3′UTR. **(D)** pmirGLO-*RNF4*-3′UTR-Mut1, pmirGLO-*RNF4*-3′UTR-Mut2 and pmirGLO-*RNF4*-3′UTR were co-transfected into ST cells with the miR-762 mimic or NC, and the relative luciferase activity was measured. **(E)** Endogenous *RNF4* mRNA levels were detected in ST cells 24 h after transfection with the miR-762 mimic or NC and the miR-762 inhibitor or inhibitor NC. **(F)** RNF4 protein levels were detected by western blotting 48 h after transfection with the miR-762 mimic or NC and the miR-762 inhibitor or inhibitor NC. Data are expressed as the means  ±  SEs of three replicates. **P < 0.01.

miR-762 was identified in our pervious study via a microarray approach as a microRNA that is expressed differentially between porcine sexually immature and mature testes^9^. miR-762 was predicted to target *RNF4* according to a RNA22 online prediction and expression pattern analysis^9^. Our further prediction showed that 2 putative miR-762-binding sites (455–476 bp and 651–677 bp) are present in the *RNF4* 3′UTR (Fig. 1A).

To ascertain the relationship between miR-762 and *RNF4*, a fragment of the *RNF4* 3′UTR containing the 2 putative miR-762-binding sites was inserted into the dual-luciferase reporter vector pmirGLO. A miR-762 mimic and a miR-762 inhibitor were synthesized to overexpress and suppress, respectively, the expression of miR-762 in ST cells (immature porcine Sertoli cells) (Supplementary Fig. S1). Reporter assay results revealed that *RNF4* is a genuine miR-762 target gene because the co-transfection of the reporter plasmid with the miR-762 mimic into ST cells reduced luciferase activity (*P* < 0.01) and co-transfection of the reporter plasmid with the miR-762 inhibitor into ST cells increased luciferase activity (*P* < 0.01) (Fig. 1B). Next, two mutated *RNF4* 3′UTR dual-luciferase reporter vectors were constructed (pmirGLO-*RNF4*-3′UTR-Mut1 contained 4-base substitutions within the first putative miR-762 seed-matched sequence in 455–476 bp, and pmirGLO-*RNF4*-3′UTR-Mut2 contained 4-base substitutions within the second putative miR-762 seed-matched sequence in 651–677 bp; Fig. 1C) and transfected into ST cells. miR-762 significantly inhibited the activity of pmirGLO-*RNF4*-3′UTR-Mut1, which indicated that miR-762 bound to the second putative binding site when the first putative binding site was destroyed. miR-762 had no apparent inhibitory effect on pmirGLO-*RNF4*-3′UTR-Mut2 (Fig. 1D), which indicated that miR-762 could not bind to the first putative binding site when the second putative binding site was destroyed. These results demonstrate that the second binding site of miR-762 (651–677 bp) in the *RNF4* 3′UTR exists.

Furthermore, endogenous *RNF4* mRNA and protein expression in ST cells were also suppressed and increased by the miR-762 mimic and miR-762 inhibitor, respectively (Fig. 1E,F). These results demonstrated that miR-762 directly targeted the *RNF4* 3′UTR.

### A spontaneous T > C mutation in the *RNF4* 3′UTR is associated with sperm quality traits

A spontaneous T > C mutation in the *RNF4* 3′UTR was detected by comparative sequencing between Large White and Chinese Meishan pigs and was named *RNF4* c.* 566 T > C according to standard mutation nomenclature^14^.

*RNF4* c.* 566 T > C was genotyped, and the allele frequencies were analysed in 466 unrelated boars from 3 different pig populations (Supplementary Table S1). The association analysis results showed that the CC boars had more semen volume per ejaculate (VOL) than the TC and TT boars in Duroc and Landrace pigs (*P* < 0.05) and that the TC boars had a higher sperm concentration (SCON) than the CC and TT boars in Large White pigs (*P* < 0.05) (Table 1).

### *RNF4* c.* 566 C creates a novel miR-762-binding site

**Figure 2:**
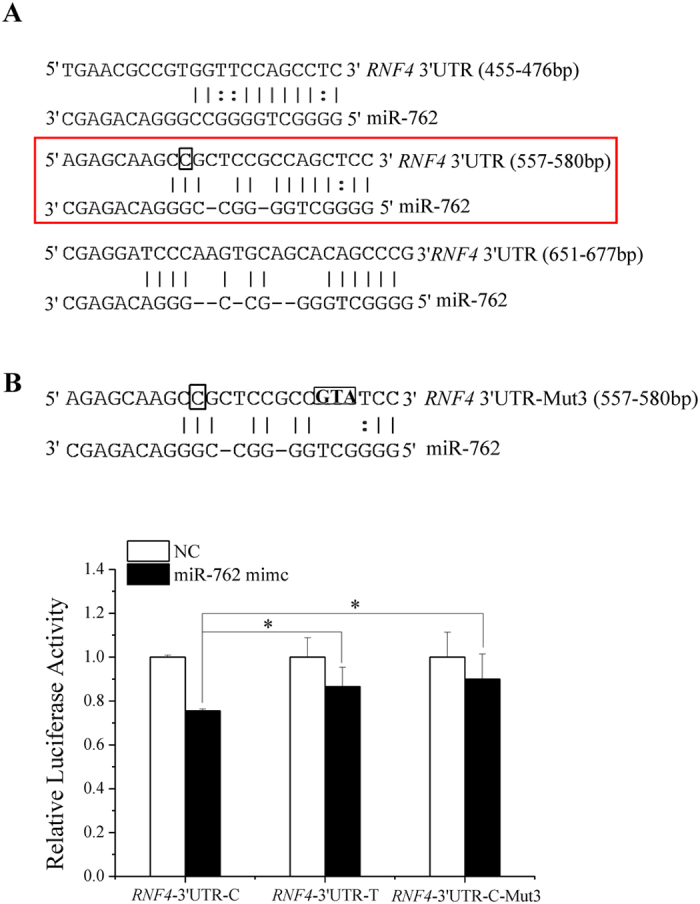
*RNF4* c.* 566 C in the *RNF4* 3′UTR creates a novel miR-762-binding site in ST cells. **(A)** For allele *RNF4* c.* 566 C, 3 binding sites for miR-762 were predicted to be present in the *RNF4* 3′UTR using RNA22. The red box indicates the novel miR-762-binding site, and the black box indicates the SNP site. **(B)** pmirGLO-*RNF4*-3′UTR-C, pmirGLO-*RNF4*-3′UTR-T and pmirGLO-*RNF4*-3′UTR-Mut3 were co-transfected with the miR-762 mimic or NC into ST cells, and relative luciferase activity was measured. Black box in the seed-matched sites indicated the sequences that were mutated to abolish the interaction between miR-762 and the novel binding site in the *RNF4* 3′UTR. *P < 0.05.

To uncover the mechanism by which *RNF4* c.* 566 T > C affects sperm quality traits, we predicted that *RNF4* c.* 566 C may lead to a novel miR-762-binding site (557–580 bp) in the *RNF4* 3′UTR through RNA22 (Fig. 2A). Three dual-luciferase reporter vectors, pmirGLO-*RNF4*-3′UTR-C, pmirGLO-*RNF4*-3′UTR-T and pmirGLO-*RNF4*-3′UTR-C-Mut3 (which contained 3-base substitutions within the putative miR-762 seed-matched sequence in 557–580 bp), were constructed and co-transfected with the miR-762 mimic or negative control (NC) into ST cells with the endogenous genotype TT at *RNF4* c.* 566 T > C locus (Supplementary Fig. S2). The relative luciferase activities of the three groups were all significantly reduced compared with the activities of the NC groups (Fig. 2B), suggesting that all three vectors contained miR-762-binding sites. The relative luciferase activity of pmirGLO-*RNF4*-3′UTR-C was significantly reduced compared with pmirGLO-*RNF4*-3′UTR-T, whereas the relative luciferase activities between pmirGLO-*RNF4*-3′UTR-C-Mut3 and pmirGLO-*RNF4*-3′UTR-T had no remarkable difference (Fig. 2B). These results demonstrated that an additional miR-762**-**binding site was created by *RNF4* c.* 566 C.

### miR-762 promotes immature Sertoli cell proliferation and inhibits apoptosis

ST cells were identified as a collection of immature porcine Sertoli cells in our previous report^15^. The number of immature Sertoli cells is important for spermatogenesis because mature Sertoli cells have no proliferative capacity and their final number depends on immature Sertoli cells^16^.

**Figure 3:**
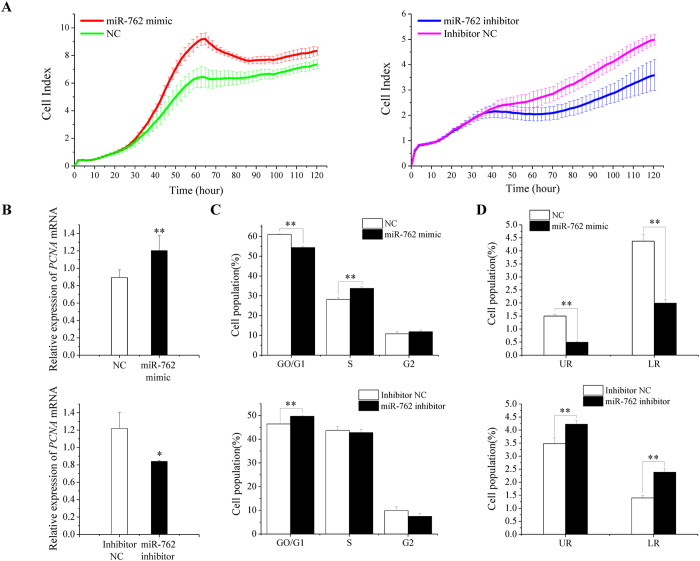
miR-762 promotes ST cell proliferation and inhibits apoptosis. ST cells were transfected with the miR-762 mimic or NC and the miR-762 inhibitor or inhibitor NC. **(A)** Cell growth dynamics were continuously monitored by the xCELLigence system. **(B)** The mRNA levels of *PCNA* (a marker gene for cell proliferation) were detected in ST cells 24 h after transfection. **(C)** Cell cycle phase was analysed 48 h after transfection by propidium iodide flow cytometry. **(D)** Cell apoptosis was measured 48 h after transfection using Annexin-V/PI staining, followed by flow cytometer analysis. UR: late apoptosis, LR: early apoptosis. **P < 0.01, *P < 0.05.

Compared with controls, miR-762-transfected cells displayed an increased growth rate, as monitored by the xCELLigence system (Fig. 3A), and showed a significantly higher mRNA expression of proliferating cell nuclear antigen (*PCNA*), which is a cell proliferation marker gene (Fig. 3B). Cell cycle analysis revealed that fewer miR-762-transfected ST cells were detected in the G0/G1 phase (quiescent resting phase/pre-DNA- synthetic phase) but more cells were detected in the S phase (DNA synthesis phase) compared with the controls (Fig. 3C and Supplementary Fig. S3), suggesting that miR-762 drives ST cells into the DNA synthesis phase (G1/S transition) and promotes cell cycle progression. The cell apoptosis assay results showed that the miR-762 mimic group had a lower apoptosis rate than the NC group (Fig. 3D and Supplementary Fig. S4). The effect of the miR-762 inhibitor on ST cell growth and apoptosis was opposite to the effect caused by the miR-762 mimic (Fig. 3A–D). All of these results demonstrated that miR-762 promoted the proliferation of immature Sertoli cells and inhibited cell apoptosis.

### RNF4 promotes immature Sertoli cell proliferation and inhibits apoptosis

**Figure 4:**
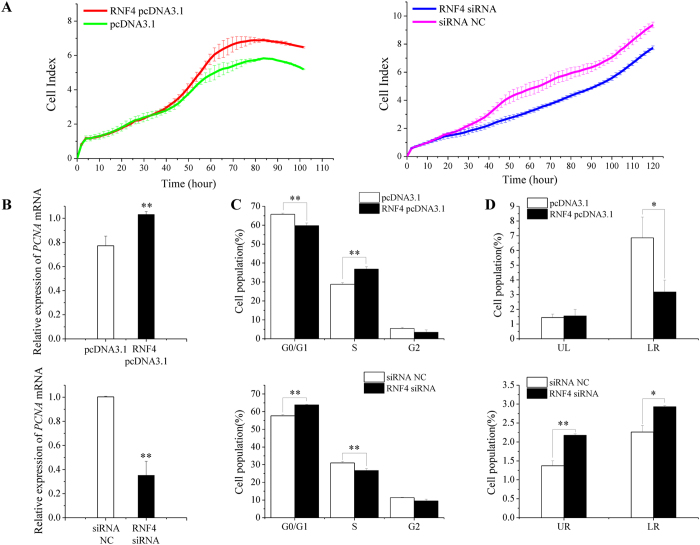
RNF4 promotes ST cell proliferation and inhibits apoptosis. ST cells were transfected with RNF4 pcDNA3.1 or pcDNA3.1 and RNF4 siRNA or siRNA NC. **(A)** Cell growth dynamics were continuously monitored by the xCELLigence system. **(B)** The *PCNA* mRNA levels were detected in ST cells 24 h after transfection. **(C)** Cell cycle phase was analysed 48 h after transfection by propidium iodide flow cytometry. **(D)** Cell apoptosis was measured 48 h after transfection using Annexin-V/PI staining, followed by flow cytometer analysis. UR: late apoptosis, LR: early apoptosis. **P < 0.01, *P < 0.05.

miR-762 was crucial for determining the porcine Sertoli cell fate by potentially regulating its target gene *RNF4*. We transfected RNF4 pcDNA3.1 and RNF4 siRNA into ST cells to overexpress and knock down RNF4, respectively (Supplementary Fig. S5). RNF4 pcDNA3.1-transfected cells displayed an increased growth rate (Fig. 4A) and *PCNA* mRNA expression level (Fig. 4B). Cell cycle analysis showed that fewer RNF4 pcDNA3.1-transfected ST cells stayed in the G0/G1 phase; however, more cells were driven into the S phase compared with the pcDNA3.1 group (Fig. 4C and Supplementary Fig. S6), suggesting that RNF4 promoted the G1/S transition. The cell apoptosis assay results showed that the RNF4 pcDNA3.1 group had a lower apoptosis rate than the control group (Fig. 4D and Supplementary Fig. S7). The effect of RNF4 siRNA on ST cell growth and apoptosis was opposite to that of RNF4 pcDNA3.1 (Fig. 4A–D). All of these results indicated that miR-762 and RNF4 exerted a similar effect on immature Sertoli cell proliferation and apoptosis.

### miR-762 reduces AR protein and AR transcriptional regulatory activity

**Figure 5:**
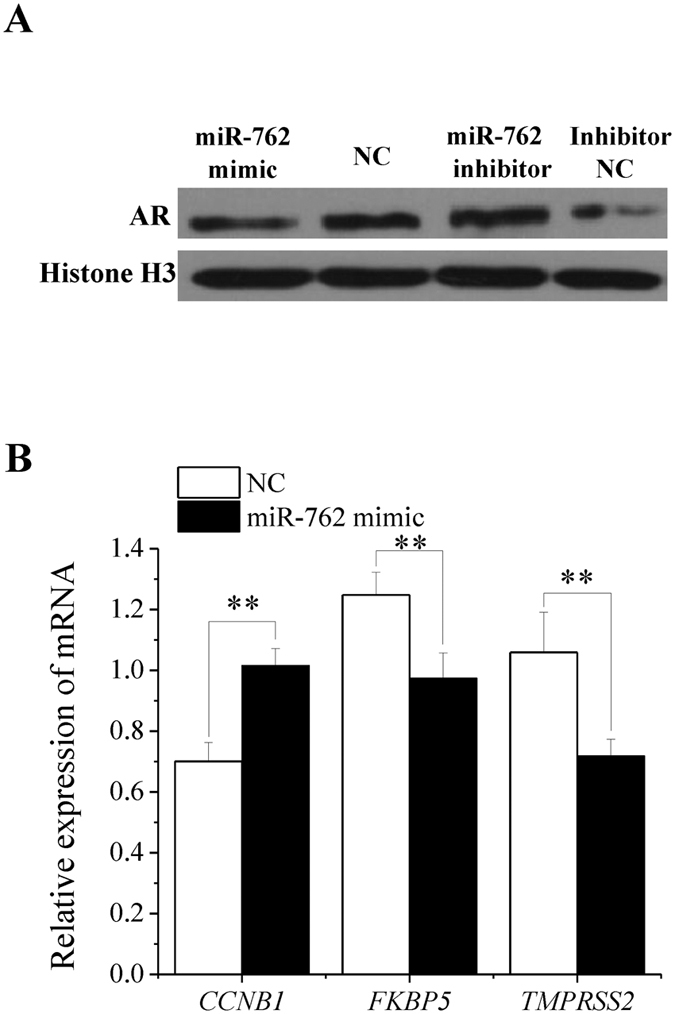
miR-762 reduces AR protein and AR transcriptional regulatory activity. **(A)** AR protein levels were detected by western blotting 48 h after transfection with the miR-762 mimic or NC and the miR-762 inhibitor or inhibitor NC in ST cells. **(B)** The mRNA expression levels of *CCNB1, FKBP5* and *TMPRSS2* were detected 24 h after transfection with the miR-762 mimic or NC. **P < 0.01.

RNF4 is a co-activator in AR-dependent transcription^17^, and AR is a nuclear transcription factor that plays a vital role in Sertoli cell growth and spermatogenesis^18^. The miR-762 mimic decreased the AR protein level in ST cell nuclei, and the miR-762 inhibitor increased the AR protein level in ST cell nuclei (Fig. 5A). In cell nuclei, AR recognizes and binds to androgen response elements (AREs) in the promoter regions of androgen-regulated genes, and it regulates the transcriptional activities of these genes^19^. The AR transcriptional regulatory activity influenced by miR-762 was checked by testing the AR-responsive genes cyclin B1 (*CCNB1*), FK506 binding protein 5 (*FKBP5*) and transmembrane protease, serine 2 (*TMPRSS2*)^20^,^21^,^22^. The miR-762 mimic up-regulated *CCNB1* mRNA, which was down-regulated by AR, and down-regulated both *FKBP5* and *TMPRSS2* mRNA, which were up-regulated by AR (Fig. 5B). The results showed that miR-762 reduced AR transcriptional regulatory activity.

### miR-762 promotes DNA damage repair

**Figure 6:**
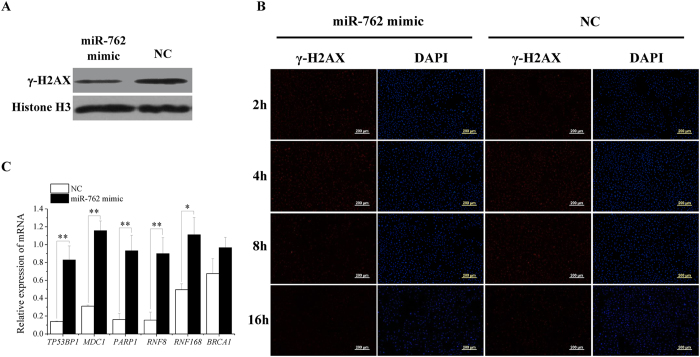
miR-762 promotes DNA damage repair. ST cells were transfected with the miR-762 mimic or NC. **(A)** After transfection for 48 h, γ-H2AX protein levels were detected using western blotting. **(B)** After transfection for 24 h, ST cells were irradiated with ultraviolet light for 20 s to cause DNA damage, γ-H2AX protein levels were tested using immunofluorescence after irradiation for 2 h, 4 h, 8 h and 16 h. γ-H2AX was positive for secondary antibody (red), and the cell nuclei were positive for DAPI (blue). Scale bar = 200 μm. **(C)** The mRNA expression levels of *TP53BP1*, *MDC1*, *PARP1*, *RNF8*, *RNF168* and *BRCA1* were detected 24 h after transfection. **P < 0.01, *P < 0.05.

DNA damage response was assessed by testing the protein level of γ-H2AX (phosphorylation of H2AX at Ser139), which is an indirect marker of unrepaired DNA breaks^23^. The miR-762 mimic reduced the γ-H2AX protein level in ST cell nuclei (Fig. 6A), indicating that miR-762 enhanced DNA damage repair or reduced DNA damage. After DNA was damaged by ultraviolet light, the γ-H2AX protein level in the miR-762 mimic group decreased faster than the γ-H2AX protein level in the NC group (Fig. 6B), indicating that miR-762 promoted the DNA damage repair response. Furthermore, DNA damage repair-related genes, including tumour protein p53 binding protein 1 (*TP53BP1*), mediator of DNA-damage checkpoint 1 (*MDC1*), poly (ADP-ribose) polymerase 1 (*PARP1*), ring finger protein 8, E3 ubiquitin protein ligase (*RNF8*), ring finger protein 168, E3 ubiquitin protein ligase (*RNF168*) and breast cancer 1, early onset (*BRCA1*)^23^,^24^, showed increased mRNA expression in the cells treated with the miR-762 mimic (Fig. 6C). This finding suggested that miR-762 promoted immature Sertoli cell proliferation and inhibited apoptosis partially due to accelerating DNA damage repair.

## Discussion

Spermatogenesis is a complex process that occurs in the seminiferous tubules of the testis. Sertoli cells, which are the only somatic cells in seminiferous tubules, play a critical role in regulating spermatogenesis because they secrete growth factors to facilitate germ cell development^25^,^26^ and provide structural supports and facilitate germ cell movement^27^. Each Sertoli cell can support limited germ cells; therefore, the Sertoli cell number in seminiferous tubules is important for germ cell production^28^. Immature Sertoli cells determine the final number of mature Sertoli cells^16^,^29^. Our results indicated that miR-762 promoted cell proliferation, inhibited cell apoptosis, and accelerated DNA damage repair in immature Sertoli cells, thus suggesting a potential function of miR-762 in pig spermatogenesis.

In the present study, miR-762 directly targeted *RNF4* 3′UTR. A SNP (*RNF4* c.* 566 T > C) in *RNF4* 3′UTR altered the number of miR-762-binding sites. Several reports revealed that SNPs within the miRNA binding sites alter the interactions between miRNAs and mRNAs and are associated with specific biological characteristics^30^,^31^,^32^. The myostatin (*MSTN*) allele of Texel sheep was characterized by a G to A transition in the 3′UTR that created a target site for miR-1 and miR-206, which caused the translational inhibition of the *MSTN* gene and contributed to the muscular hypertrophy of Texel sheep^33^. *RNF4* c.* 566 T > C was significantly associated with sperm quality traits in 3 pig populations, possibly due to changing the binding status between miR-762 and *RNF4* 3′UTR. The genetic effect of *RNF4* c.* 566 T > C on SCON is different among the breeds, which is possibly caused by different genetic backgrounds of the three pig breeds.

**Figure 7:**
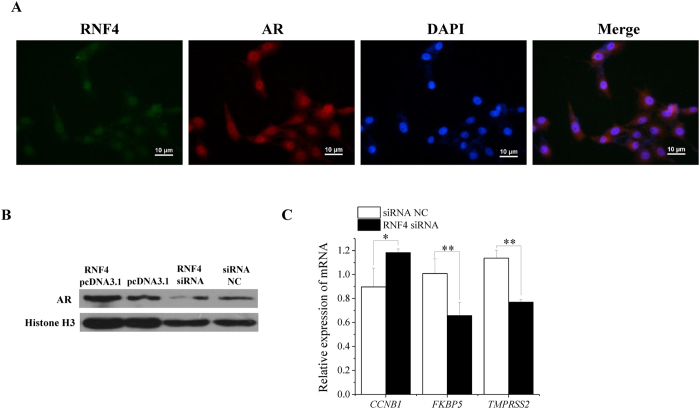
RNF4 increases AR protein in ST cell nuclei and regulates AR transcriptional regulatory activity. **(A)** Immunofluorescence was applied to verify the protein-protein interaction between RNF4 and AR, and the ST cells were immunostained by anti-RNF4 (green) and anti-AR (red) antibody. Cell nuclei were positive for DAPI (blue). Scale bar = 10 μm. **(B)** AR protein levels were detected 48 h after transfection with RNF4 pcDNA3.1 or pcDNA3.1 and RNF4 siRNA or siRNA NC in ST cells. **(C)** The mRNA expression levels of *CCNB1, FKBP5* and *TMPRSS2* were detected 24 h after transfection with RNF4 siRNA or siRNA NC. **P < 0.01, *P < 0.05.

RNF4 is a nuclear transcription factor that acts as a co-activator in AR-dependent transcription^17^. In our study, RNF4 co-localized with AR in ST cell nuclei, as observed by immunofluorescence (Fig. 7A). In addition, the AR protein levels increased and decreased when RNF4 was overexpressed and inhibited in ST cells, respectively (Fig. 7B). Furthermore, decreased RNF4 protein weakened the AR transcriptional regulatory activity (Fig. 7C), which was consistent with the effect of miR-762 on AR transcriptional regulatory activity (Fig. 5B). These results demonstrated that miR-762 affected immature Sertoli cells by partially weakening the AR transcriptional regulatory activity caused by decreased RNF4.

AR is a nuclear receptor well known for its role in spermatogenesis as AR is required for complete meiosis, adhesion of spermatids, spermiation and blood-testis barrier formation^18^,^34^. The overexpression of AR in immature Sertoli cells caused the cells to become premature and decreased the final mature Sertoli cell number, resulting in a reduced final germ cell number^35^. AR inactivation by a daily oral dose of an androgen receptor antagonist beginning at one-week of age caused porcine Sertoli cell proliferation^36^. In this study, miR-762 promoted immature Sertoli cell proliferation and inhibited apoptosis, which was possibly due to lower AR expression and lower transcriptional regulatory activity. Although Sertoli cell-specific AR-knockout male mice were infertile, the Sertoli cell number was normal^18^. These findings indicated that low AR levels in immature Sertoli cells could maintain cell proliferation and inhibit cell differentiation at a specific phase.

miR-762 accelerated DNA damage repair in ST cells and caused the cells to quickly engage in normal DNA replication and division, and this finding supported both the cell cycle results demonstrating that miR-762 promoted ST cell transformation from G0/G1 phase to S phase and the cell proliferation results showing that miR-762 promoted ST cell proliferation.

**Figure 8:**
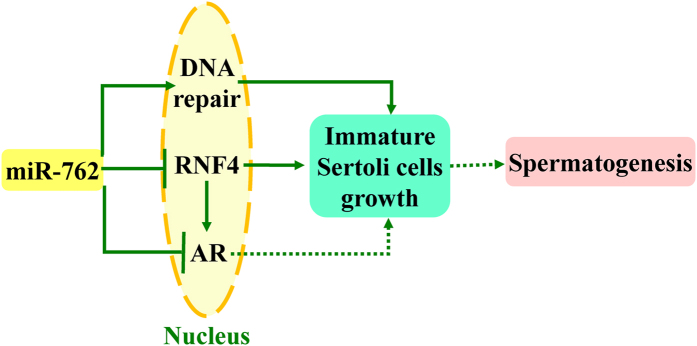
Model of the main findings of this study. miR-762 promotes immature Sertoli cells proliferation and inhibits apoptosis by inhibiting RNF4 and AR and by enhancing DNA damage repair capacity, and miR-762 is speculated to function in pig spermatogenesis.

In summary, our study showed that miR-762 promotes porcine immature Sertoli cell proliferation and inhibits apoptosis in a comprehensive way by regulating RNF4, AR and DNA damage repair genes (Fig. 8). We speculate that miR-762 functions in pig spermatogenesis by determining the fate of immature Sertoli cells.

## Materials and Methods

### Animals

All animal procedures were performed according to protocols approved by the Biological Studies Animal Care and Use Committee of Hubei Province, PR China. All of the studies involving animals were conducted according to the regulation (No. 5 proclamation of the Standing Committee of Hubei People’s Congress) approved by the Standing Committee of Hubei People’s Congress, PR China. The sample collection was approved by the Ethics Committee of Huazhong Agricultural University with the permit number No. 30700571. The animals used for genotyping and association analysis included Duroc (n = 186), Large White (n = 123) and Landrace (n = 157) pigs, with sperm quality records including semen volume per ejaculate (VOL), sperm concentration (SCON), sperm motility (MOT) and abnormal sperm rate (ASR). The animals were allowed access to food and water *ad libitum* under normal conditions.

### SNP analysis

Based on the *sus scrofa RNF4* mRNA sequence (NM_001044528.2), primers RNF4-SNP-PF and RNF4-SNP-PR (Supplementary Table S3) were designed to amplify the 3′UTR of the *RNF4* gene. The amplified sequences of a Large White pig and a Chinese Meishan pig were aligned using Clustalw2 (http://www.ebi.ac.uk/Tools/msa/clustalw2/) to detect the SNP. Boars were genotyped for *RNF4* c.* 566 T > C in the 3′UTR by PCR-RFLP. A primer pair (RNF4-SNP-PF and RNF4-SNP-PR, Supplementary Table S3) was used to amplify a specific fragment of *RNF4* 3′UTR, the restriction enzyme *Mbi*I (Thermo Scientific, Waltham, MA, USA) was used to digest the PCR products, and the digested products were separated by electrophoresis on a 2% agarose gel. Population association analysis was performed as previously described^37^. Briefly, the relationships between genotypes and sperm quality traits were assessed using the general linear model (GLM) procedure of SAS version 9.2, and the additive and dominance effects were estimated using the REG procedure of SAS version 9.2.

### Cell culture and transfection

The swine testicular (ST) cell line (ATCC^®^ CRL-1746™) was isolated from swine foetal testes of 80 to 90 day pigs and was identified as a collection of immature porcine Sertoli cells in our previous report^15^. ST cells were cultured in high-glucose Dulbecco’s modified Eagle’s medium (HyClone, Logan, Utah, USA) with 10% (v/v) foetal bovine serum (Gibco, Grand Island, NY, USA) at 5% CO2 and 37 °C. The cells were transfected with the miR-762 mimic, negative control (NC), miR-762 inhibitor and inhibitor NC (Supplementary Table S2) (GenePharma, Shanghai, China) using Lipofectamine 2000 transfection reagent (Invitrogen, Carlsbad, CA, USA). Opti-MEM I Reduced Serum Medium (Gibco, Grand Island, NY, USA) was used to dilute Lipofectamine 2000 and nucleic acids.

### Dual-luciferase reporter assays

The 3′UTR of the pig *RNF4* gene was amplified by reverse transcription-polymerase chain reaction (RT-PCR) with the primer pair RNF4-3′UTR-PF and RNF4-3′UTR-PR (Supplementary Table S3) using total RNA extracted from untreated ST cells with the HP Total RNA Kit (Omega Bio-tek, Norcross, GA, USA). The PCR products were directionally cloned into the pmirGLO dual-luciferase miRNA target expression vector (Promega, Madison, WI, USA). Site-directed mutagenesis in the seed regions of the putative miR-762-binding sites in the *RNF4* 3′UTR were generated using overlap-extension PCR with mutagen primer pairs (RNF4-3′UTR-Mut1-PF and RNF4-3′UTR-Mut1-PR, RNF4-3′UTR-Mut2-PF and RNF4-3′UTR-Mut2-PR, RNF4-3′UTR-Mut3-PF and RNF4-3′UTR-Mut3-PR; Supplementary Table S3). ST cells were co-transfected with the 3′UTR luciferase reporter vectors and the miR-762 mimic or miR-762 inhibitor. After 24 h of transfection, luciferase activity was measured using the Dual-Glo Luciferase Assay System (Promega, Madison, WI, USA). Firefly luciferase activity was normalized to the corresponding Renilla luciferase activity.

### RNF4 pcDNA3.1-expression vector and RNF4 siRNA

Porcine *RNF4* CDS was amplified by RNF4-pcDNA3.1-PF and RNF4-pcDNA3.1-PR primers (Supplementary Table S3) and inserted into pcDNA3.1 vector to construct an *RNF4* gene overexpression vector. RNF4 double-stranded siRNAs (Supplementary Table S2) were obtained from GenePharma (Shanghai, China).

### Quantitative (Q)-PCR

Total RNA was extracted from ST cells with the HP Total RNA Kit (Omega Bio-tek, Norcross, GA, USA) and was treated with DNase I (Thermo Scientific, Waltham, MA, USA). The concentration and quality of RNA were assessed with the NanoDrop 2000 (Thermo Scientific, Waltham, MA, USA) and agarose gel electrophoresis. Total RNA was reverse transcribed using the Revert Aid First Strand cDNA Synthesis Kit (Thermo Scientific, Waltham, MA, USA). For miR-762 quantification, stem-loop RT-PCR was executed with U6 small nuclear RNA (snRNA) as an internal control. All primers were designed based on miR-762 sequence collected from MirBase release 21 (http://www.mirbase.org/). RT-PCR was used to quantify mRNA (*RNF4*, *CCNB1*, *FKBP5*, *TMPRSS2*, *TP53BP1*, *BRCA1*, *MDC1*, *PARP1*, *RNF8*, *RNF168*, *PCNA*) with *β-actin* as an internal control. qPCR was performed in triplicate using iQ SYBR green Supermix (Bio-Rad, Hercules, CA, USA) and the LightCycler^®^480 (Roche Applied Science, Penzberg, Upper Bavaria, Germany).

### Western blotting

Whole-cell protein and cell nuclear protein were extracted using RIPA buffer (Beyotime, Haimen, China) and the Nuclear and Cytoplasmic Protein Extraction Kit (Beyotime, Haimen, China), respectively. Sodium dodecyl sulphate-polyacrylamide gel electrophoresis (SDS-PAGE) was performed, followed by protein transfer to polyvinylidene fluoride membrane (Merk Millipore, Billerica, MA, USA). Primary antibodies for RNF4 (Santa Cruz, CA, USA), AR (BIOSS, Beijing, China), Phospho-Histone H2A.X (Ser139) (Cell Signalling Technology, Danvers, MA, USA) and Histone H3 (Wuhan Guge, Wuhan, China) and secondary antibodies for HRP-labelled rabbit anti-goat IgG (Wuhan Guge, Wuhan, China), HRP-labelled goat anti-rabbit IgG (Wuhan Guge, Wuhan, China) and HRP-labelled goat anti-mouse IgG (Wuhan Guge, Wuhan, China) were used to detect RNF4, AR, γ-H2AX and Histone H3 protein expression.

### Immunofluorescence

Immunofluorescence was performed as previously described^15^. The primary antibodies included anti-RNF4 antibody (Santa Cruz, CA, USA), anti-AR antibody (BIOSS, Beijing, China) and anti-Phospho-Histone H2A.X (Ser139) antibody (Cell Signalling Technology, Danvers, MA, USA). The secondary antibodies included donkey anti-goat FITC-IgG (Wuhan Guge, Wuhan, China), goat anti-rabbit Cy3-IgG (Wuhan Guge, Wuhan, China) and goat anti-rabbit FITC-IgG (Wuhan Guge, Wuhan, China).

### DNA damage repair analysis

After transfection with the miR-762 mimic and NC for 24 h, ST cells were irradiated with ultraviolet light (253.7 nm wavelength) for 20 s to cause DNA damage. DNA damage repair response was checked by testing γ-H2AX protein levels via immunofluorescence after irradiation for 2 h, 4 h, 8 h and 16 h.

### Cell proliferation analysis

ST cells were seeded on a 16-well E-Plate with 5000 cells per well and allowed to grow for 12–24 h. The cells were transfected with miR-762 mimic or miR-762 inhibitor when the cell index reached 1.0–2.0 with three wells per treatment. Cell growth and proliferation were monitored by an xCELLigence RTCA DP instrument (Roche Applied Science, Penzberg, Upper Bavaria, Germany).

The cell cycle was analysed with the Cell Cycle Detection Kit (KeyGEN BioTECH, Nanjing, China). Briefly, 48 h after transfection, the ST cells were fixed in 70% (v/v) ethanol overnight at −20 °C. Following incubation in 50 mg/ml propidium iodide (PI) for 30 min at 4 °C, the cells were analysed using FACSCalibur Flow Cytometry (Becton Dickinson, Franklin Lakes, NJ, USA) and the ModFit software (Verity Software House). The proliferative index was derived by estimating the proportion of mitotic cells from a total of 20,000 cells.

### Cell apoptosis analysis

Cell apoptosis was analysed using the Annexin V-FITC Apoptosis Detection Kit (KeyGEN BioTECH, Nanjing, China). Briefly, the ST cells were harvested through trypsinization (without EDTA) and washed twice with phosphate-buffered saline (HyClone, Logan, Utah, USA). Following centrifugation at 2000 rpm for 5 min, the sediment was re-suspended in 500 μl Binding Buffer and incubated with 5 μl FITC-conjugated Annexin V and 5 μl PI for 15 min. The samples then were analysed by FACSCalibur Flow Cytometry (Becton Dickinson, Franklin Lakes, NJ, USA).

### Bioinformatics method and statistical analysis

The miR-762 targets were predicted by a computer-aided algorithm RNA22 (https://cm.jefferson.edu/rna22/Interactive/). All results are presented as the mean  ±  standard error (SE), based on at least three replicates for each treatment. Two-tailed Student’s t-tests were used for P-value calculations.

## Additional Information

**How to cite this article**: Ma, C. *et al*. miR-762 promotes porcine immature Sertoli cell growth via the ring finger protein 4 (*RNF4*) gene. *Sci. Rep.* **6**, 32783; doi: 10.1038/srep32783 (2016).

## Supplementary Material

Supplementary Information

## Figures and Tables

**Table 1:** Association between *RNF4* c.*566 T > C and boar sperm quality traits in 3 pig populations.

Populations	Traits	Genotype (μ ± SE)			Effect (μ ± SE)	
		CC	TC	TT	Additive	Dominance
Duroc	*N*	135	44	7		
	VOL (ml)	197.90 ± 2.76^**a**^	186.75 ± 4.83^**b**^	178.29 ± 12.11	9.80 ± 6.21	0.67 ± 3.93
	SCON (10^8/^ml)	2.67 ± 0.05	2.59 ± 0.10	3.00 ± 0.24	−0.16 ± 0.12	0.12 ± 0.08
	MOT (%)	86.10 ± 0.37	86.55 ± 0.65	88.14 ± 1.63	−1.02 ± 0.84	0.29 ± 0.53
	ASR (%)	8.55 ± 0.20	8.45 ± 0.35	9.71 ± 0.87	−0.58 ± 0.44	0.34 ± 0.28
Large White	*N*	7	36	80		
	VOL (ml)	197.00 ± 20.01	190.72 ± 8.82	193.78 ± 5.92	32.70 ± 26.16	30.48 ± 15.70
	SCON (10^8/^ml)	2.15 ± 0.35^**b**^	3.12 ± 0.15^**a**^	2.69 ± 0.10^**b**^	−1.36 ± 0.46^**^	−0.87 ± 0.28^**^
	MOT (%)	89.57 ± 1.68	87.56 ± 0.74	86.54 ± 0.50	4.07 ± 2.24	1.52 ± 1.34
	ASR (%)	8.66 ± 1.21	7.89 ± 0.53	7.32 ± 0.36	−1.11 ± 1.54	1.12 ± 0.93
Landrace	*N*	26	79	52		
	VOL (ml)	199.00 ± 11.58^**a**^	190.53 ± 6.64	184.81 ± 8.19^**b**^	7.10 ± 7.09	0.69 ± 4.86
	SCON (10^8/^ml)	2.52 ± 0.22	2.25 ± 0.13	2.47 ± 0.16	0.03 ± 0.14	0.12 ± 0.09
	MOT (%)	84.46 ± 1.34	84.77 ± 0.77	84.56 ± 0.95	−0.05 ± 0.82	−0.13 ± 0.56
	ASR (%)	5.92 ± 0.66	6.28 ± 0.38	6.21 ± 0.47	−0.15 ± 0.41	−0.11 ± 0.28

*Note*: VOL: semen volume per ejaculate; SCON: sperm concentration; MOT: sperm motility; ASR: abnormal sperm rate. a and b, *P* < 0.05; **P < 0.05. *N*: Number of boars.
