# Supplementary material for: miR-762 promotes porcine immature Sertoli cell growth via the ring finger protein 4 (RNF4) gene
Source: Sci Rep. 2016 Sep 6;6:32783. doi: 10.1038/srep32783 (PMC5011707; doi:10.1038/srep32783)
Supplement: Supplementary Information [file srep32783-s1.pdf]

miR-762 promotes porcine immature Sertoli cell growth via the ring finger protein 4 (*RNF4*) gene

Changping Ma[1], Huibin Song[1], Lei Yu[1], Kaifeng Guan[1], Pandi Hu[1], Yang Li[1], Xuanyan Xia[2], Jialian Li[1], Siwen Jiang[1, 3], Fenge Li[1, 3*]

[1]Key Laboratory of Pig Genetics and Breeding of Ministry of Agriculture & Key Laboratory of Agricultural Animal Genetics, Breeding and Reproduction of Ministry of Education, Huazhong Agricultural University, Wuhan 430070, PR China

[2]College of Informatics, Huazhong Agricultural University, Wuhan 430070, PR China

[3]The Cooperative Innovation Center for Sustainable Pig Production, Wuhan 430070, PR China

* lifener@mail.hzau.edu.cn

Supplementary Table S1: Genotype and allele frequencies of *RNF4* c.* 566 T>C in 3 pig populations

Populations	No. pigs	No. genotypes			Allele frequency	
		CC	TC	TT	C	T
Duroc	186	135	44	7	0.84	0.16
Large White	123	7	36	80	0.20	0.80
Landrace	157	26	79	52	0.42	0.58

Supplementary Table S2: Synthetic oligo sequences

Name	Sequence (5'-3')
miR-762 mimic	GGGGCUGGGGCCGGGACAGAGC
	GCUCUGUCCCGGCCCCAGCCCC
negtive control (NC)	UUCUCCGAACGUGUCACGUTT
	ACGUGACACGUUCGGAGAATT
miR-762 inhibitor	GCUCUGUCCCGGCCCCAGCCCC
Inhibitor NC	CAGUACUUUUGUGUAGUACAA
RNF4 siRNA	GACAUCACCUGUGAAUCUUTT
	AAGAUUCACAGGUGAUGUCTT
siRNA NC	UUCUCCGAACGUGUCACGUTT
	ACGUGACACGUUCGGAGAATT

Supplementary Table S3: Primer sequences

Name	Sequence (5'-3')
RNF4-SNP-PF	ATTTGCTGCGACGTTTGC
RNF4-SNP-PR	GATGGACCTTGTCTGCCTTG
RNF4-QPCR-PF	AGCAGTGATGCCGAGGAGC
RNF4-QPCR-PR	AATGGGGTGGTATCGTTTGTG
CCNB1-QPCR-PF	TTGACTGGCTAGTGCAGGTTC
CCNB1-QPCR-PR	CTGGAGGGTACATTTCTTCATA
FKBP5-QPCR-PF	AGGAATGAACCGTTTGTCTT
FKBP5-QPCR-PR	CCTTCGTTGGGATTTGAGTA
TMPRSS2-QPCR-PF	ATACACATCTGCGGAGGC
TMPRSS2-QPCR-PR	TGAAGGTCATTGGCGTCT
TP53BP1-QPCR-PF	AGCCAAATAGGACAAGCAGT

TP53BP1-QPCR-PR	ATCTTCAGCACCAACGGAGT
BRCA1-QPCR-PF	CACAGCAGCAAATACAACC
BRCA1-QPCR-PR	TCTCAGTAGCCGTCAAATC
MDC1-QPCR-PF	GGACCCAGTTCCTTAGCA
MDC1-QPCR-PR	CCCTCTTTGGAGCCGTTT
PARP1-QPCR-PF	AGTATGCCAAGTCCAACAGAA
PARP1-QPCR-PR	CCATCTACCTCGTCACCTTT
RNF8-QPCR-PF	AGCAATCATTCACGCCAAGA
RNF8-QPCR-PR	ACATTCCGCATCATCCAC
RNF168-QPCR-PF	TCAGAATGCCAGTGCCAGAT
RNF168-QPCR-PR	CTCCGATTGTTGCCCAGA
β -actin-QPCR-PF	CCAGGTCATCACCATCGG
β -actin-QPCR-PR	CCGTGTTGGCGTAGAGGT
PCNA-QPCR-PF	ACCGCTGCGACCGCAATTTG
PCNA-QPCR-PR	ACGTGCAAATTCACCAGAAGGCATC
miR-762-RT-loop	GTCGTATCCAGTGCAGGGTCCGAGGTATTCGCACT GGATACGACGCTCTGTC
miR-762-QPCR-PF	CGGGGCAGGGACAGAG
miR-762-QPCR-PR	GTGCAGGGTGCGAGGT
U6-QPCR-PF	GCTTCGGCAGCACATATACT
U6-QPCR-PR	TTCACGAATTTGCGTGTCAT
RNF4-pcDNA3.1-PF	CTAGCTAGCGCCACCATGAGCACGAGAAAGCGTCG
RNF4-pcDNA3.1-PR	CCCAAGCTTTCATATATAAATGGGGTGGT
RNF4-3'UTR-PF	GGGTTTAAACAGAACGCTAACACTTGCC
RNF4-3'UTR-PR	CCCTCGAGAGAACGGGCTTTATGGCA
RNF4-3'UTR-Mut1-PF	GCCGTGGTTCCTATGTCCGTCCAGA
RNF4-3'UTR-Mut1-PR	TCTGGACGGACATAGGAACCACGGC
RNF4-3'UTR-Mut2-PF	CTGCTCCGCCTAAGCCTGTGGGC
RNF4-3'UTR-Mut2-PR	GCCCACAGGCTTAGGCGGAGCAG
RNF4-3'UTR-Mut3-PF	AGTGCAGCACGTATCGAAGCGACC
RNF4-3'UTR-Mut3-PR	GGTCGCTTCGATACGTGCTGCACT

Note: PF was upstream primer. PR was downstream primer.

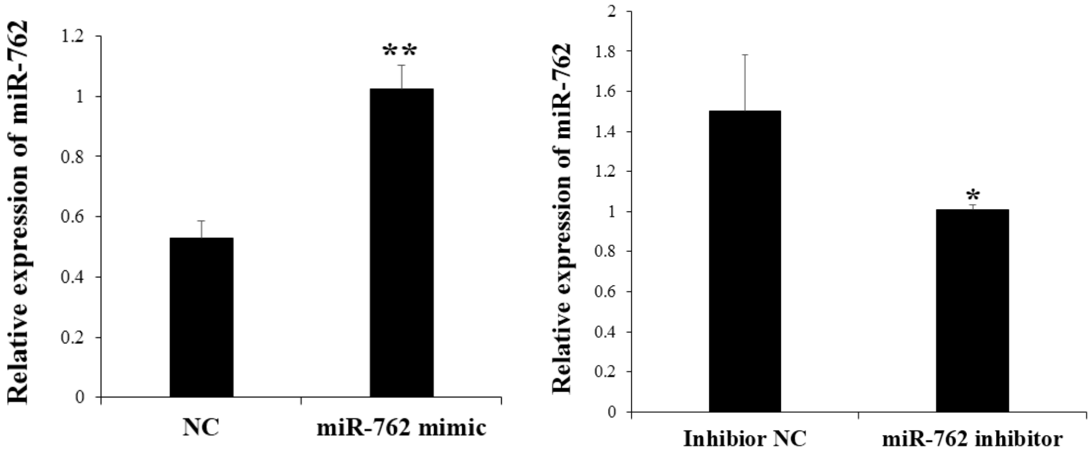

Supplementary Figure S1. miR-762 expression was detected in ST cells 24 h after transfecting with the miR-762 mimic or NC and the miR-762 inhibitor or inhibitor NC. $**P < 0.01$, $*P < 0.05$.

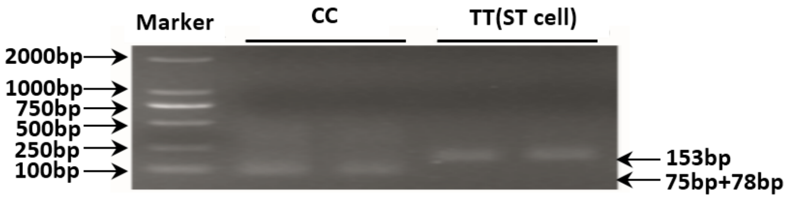

Supplementary Figure S2. Genotyping for *RNF4* c.* 566 T>C in ST cells using PCR-*Mbi*I-RFLP. Genotype TT: 153 bp; Genotype CC: 75 bp+78 bp. Marker: DNA molecular marker DL 2,000.

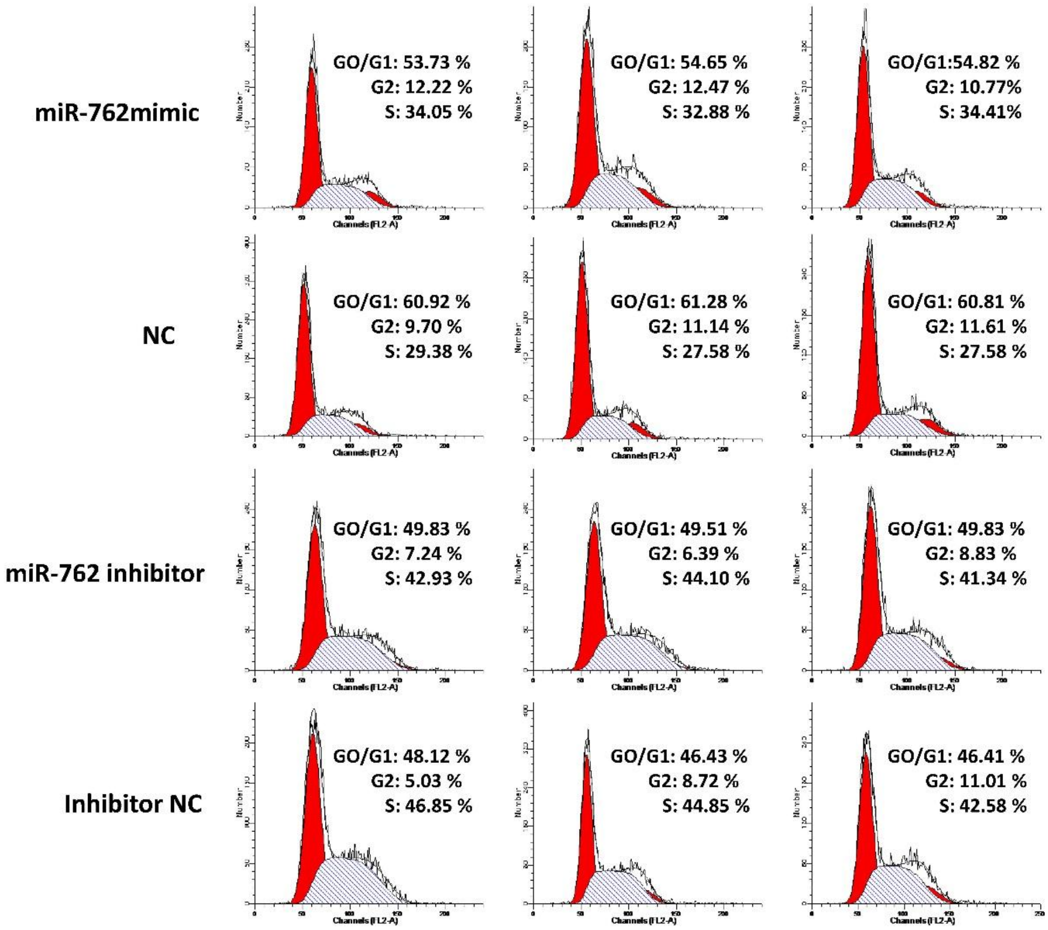

Supplementary Figure S3. The histograms of cell cycle analyzed by flow cytometry.

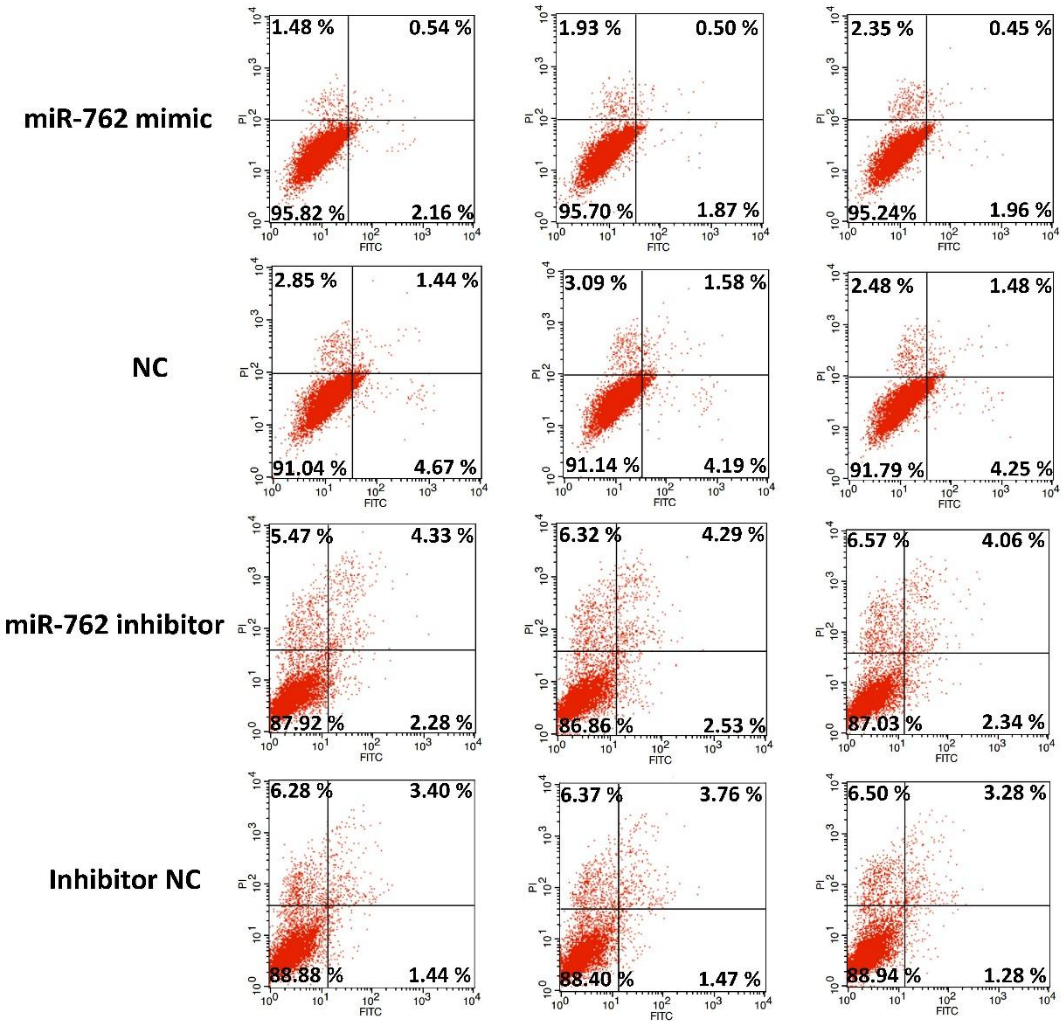

Supplementary Figure S4. The histograms of cell apoptosis analyzed by flow cytometry.

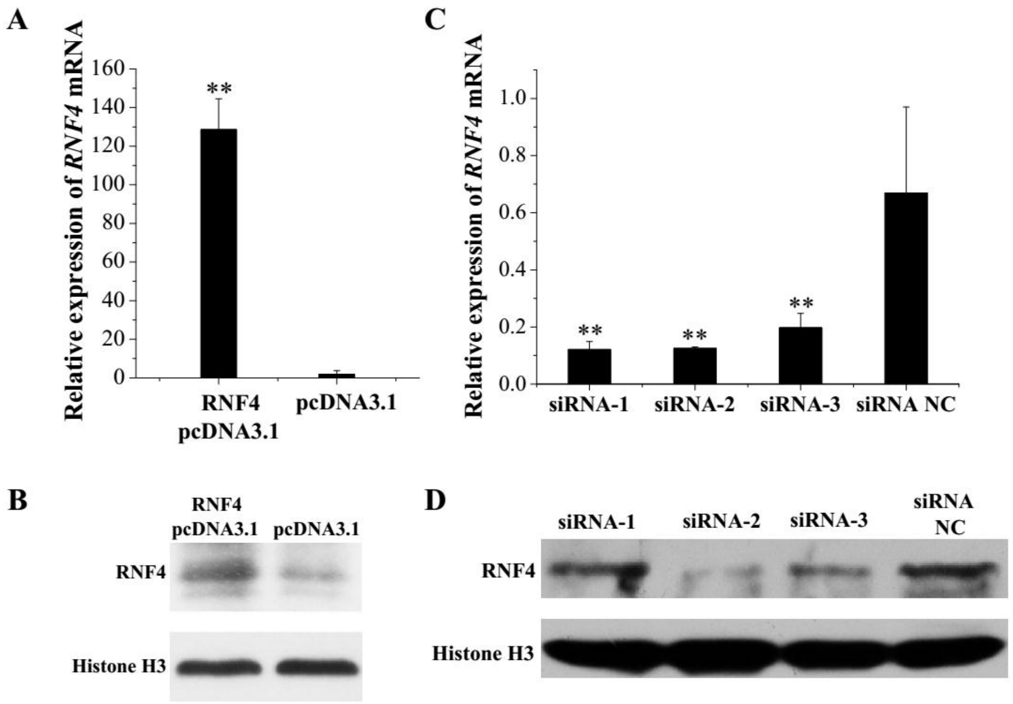

Supplementary Figure S5. RNF4 pcDNA3.1 was transfected into ST cells to overexpress RNF4 mRNA (**A**) and protein (**B**) levels. siRNAs were transfected into ST cells to inhibit RNF4 mRNA (**C**) and protein (**D**) levels. siRNA-2 was chosen to use in the following experiments.

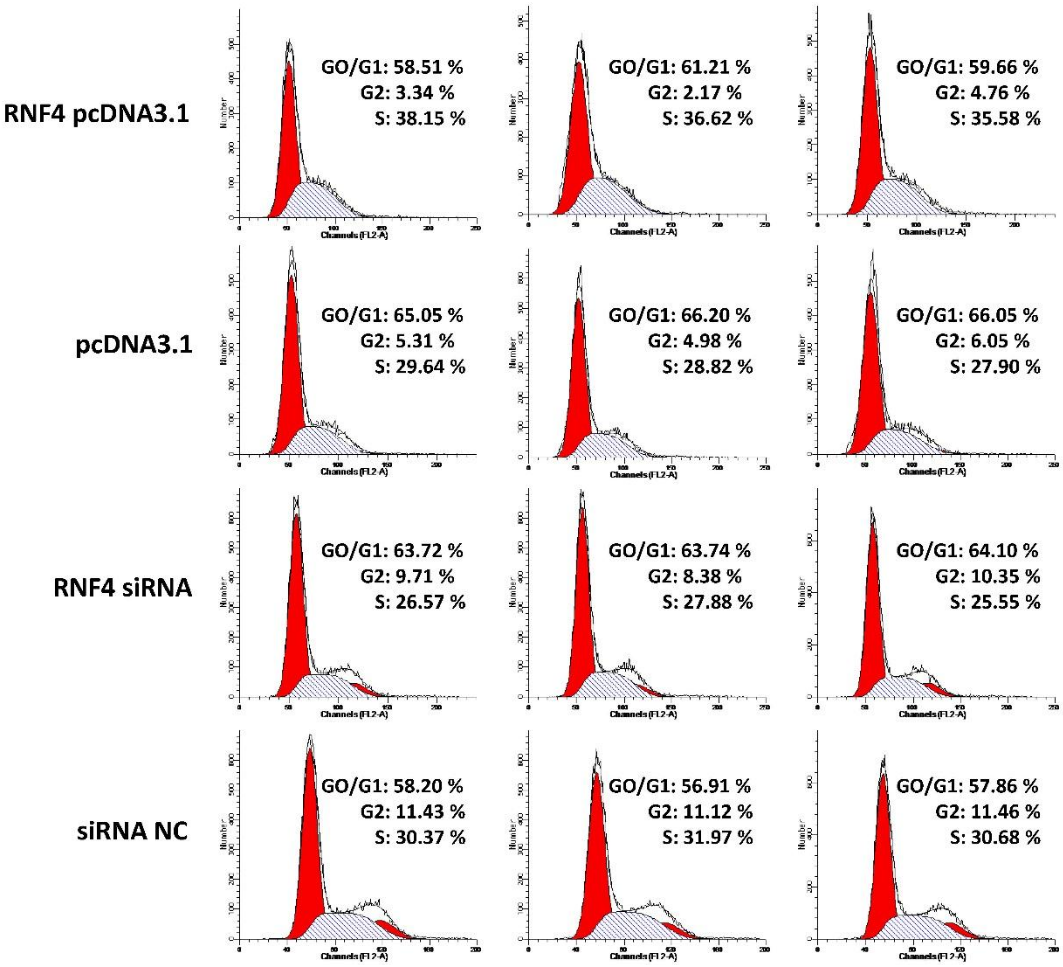

Supplementary Figure S6. The histograms of cell cycle analyzed by flow cytometry.

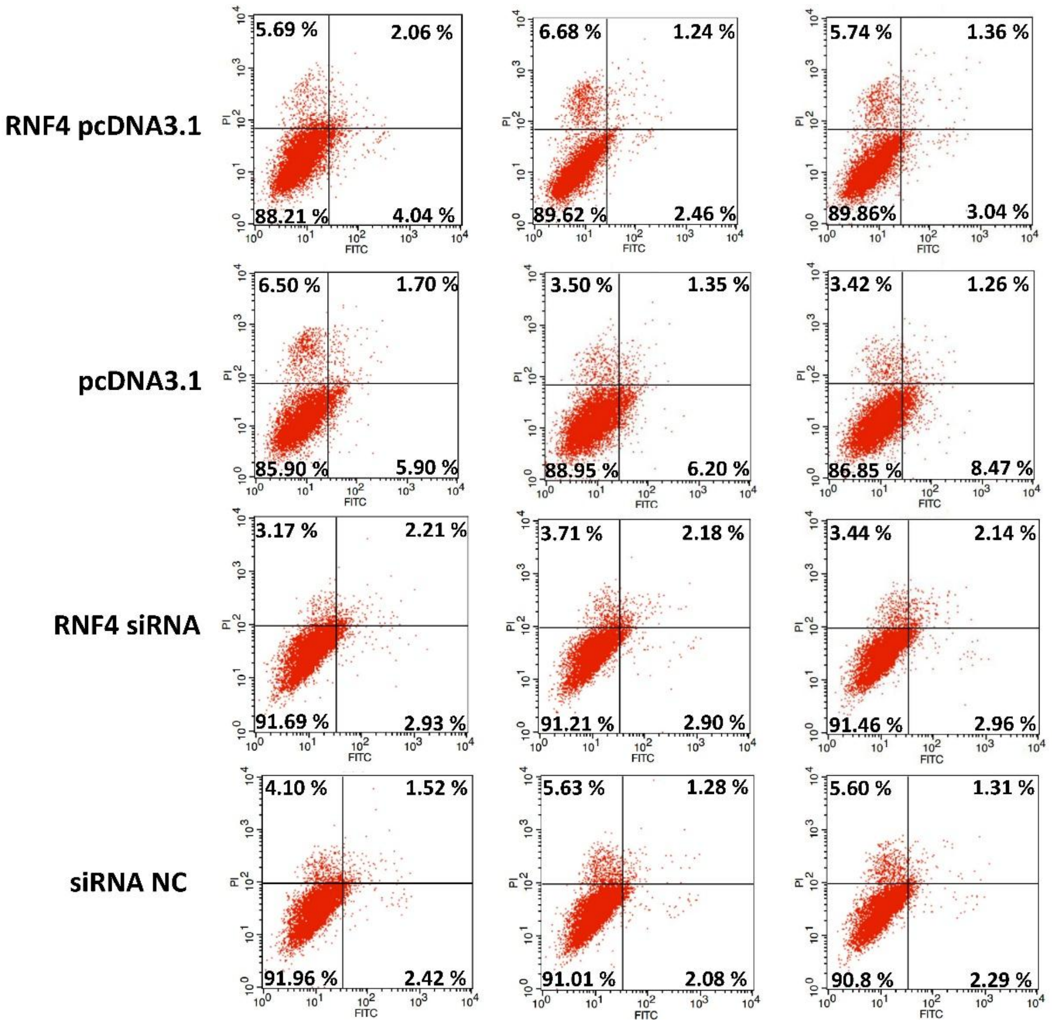

Supplementary Figure S7. The histograms of cell apoptosis analyzed by flow cytometry.
